# Orphan drug propranolol for infantile hemangioma: ten-year real-world safety data from the FAERS database

**DOI:** 10.1186/s13023-026-04331-4

**Published:** 2026-04-01

**Authors:** Xuanjia Li, Xingyu Zhao, Chengzhe Yang, Anwei Chen

**Affiliations:** 1 Department of Plastic, Aesthetic, and Burn Surgery, Qilu Hospital of Shandong University, Jinan, Shandong 250012 China; 2 Department of Oral and Maxillofacial Surgery, Qilu Hospital of Shandong University, Jinan, Shandong 250012 China

**Keywords:** Propranolol, Infantile hemangioma, Adverse events, Pharmacovigilance, FAERS

## Abstract

**Objective:**

Propranolol, a non-selective β-adrenergic receptor blocker, is the first FDA-approved orphan drug for the treatment of infantile hemangioma (IH). While it has demonstrated substantial therapeutic benefits, the safety profile of propranolol in infants remains insufficiently characterized, particularly in real-world settings.

**Methods:**

A retrospective pharmacovigilance analysis of FAERS reports from Q1 2015 to Q4 2024 was conducted, applying four signal detection algorithms (ROR, PRR, BCPNN, MGPS) to identify disproportional AE signals related to propranolol in IH.

**Results:**

A total of 1615 AE reports were included. Signal detection identified events across 20 System Organ Classes (SOCs), with vascular, psychiatric, and metabolism and nutrition disorders being most prominent. At the Preferred Term (PT) level, the most frequently reported AEs were sleep disorder (*n* = 149, ROR = 32.67), peripheral coldness (*n* = 87, ROR = 108.73), and hypoglycaemia (*n* = 62, ROR = 22.95), which were pharmacologically plausible and clinically relevant.

**Conclusions:**

This study identified safety signals associated with propranolol in infants with IH, highlighting risks across organ systems. These findings support the need for vigilant monitoring, individualized risk assessment, and refinement of labeling recommendations to optimize safety in this vulnerable population.

**Supplementary information:**

The online version contains supplementary material available at 10.1186/s13023-026-04331-4.

## Introduction

Infantile hemangioma (IH) is the most common benign vascular tumor of infancy, characterized by abnormal proliferation of vascular endothelial cells and an imbalance between cell growth and apoptosis. IH typically involves the skin and subcutaneous tissues, with a predilection for the head and neck, followed by the trunk and extremities [1]. The reported incidence ranges from 2% to 10%, with higher prevalence in Caucasian females and preterm infants [2]. Clinically, superficial IHs appear as bright red plaques or patches, whereas deep lesions present as bluish or nodular masses [3]. While most IHs undergo spontaneous involution, approximately 10–15% require medical intervention due to complications such as disfigurement, visual axis obstruction, airway compromise, or high-output cardiac failure [4]. Therapeutic strategies for IH include watchful waiting, pharmacologic treatment—primarily β-adrenergic receptor blockers—laser therapy, and surgical intervention [5, 6]. In refractory cases, adjunctive intralesional sclerotherapy has demonstrated potential as a supplementary approach [7].

Propranolol, a classical non-selective β-adrenergic receptor antagonist, was originally developed for cardiovascular conditions such as hypertension and arrhythmias. In 2008, it was serendipitously discovered to be effective against IH, and subsequently became the first-line systemic therapy for this condition [8]. In 2014, the U.S. Food and Drug Administration (FDA) approved an oral pediatric formulation (Hemangeol®) specifically for IH, making propranolol the first orphan drug formally approved for this indication. Its mechanisms of action include vasoconstriction, downregulation of angiogenic factors such as vascular endothelial growth factor (VEGF) and basic fibroblast growth factor (bFGF), and induction of endothelial cell apoptosis, collectively promoting lesion regression [9]. Clinical trials have reported complete or near-complete response rates in up to 60% of treated infants, substantially improving long-term outcomes [10]. As an FDA-designated orphan drug, propranolol was granted seven years of marketing exclusivity from 2014 to 2021. While this designation has incentivized drug development for rare pediatric conditions, it also underscores the need for robust post-marketing pharmacovigilance—especially given the limited pre-approval safety data in infants, a highly vulnerable population.

Despite its well-documented efficacy, the safety profile of propranolol in infants remains incompletely characterized. Reported adverse events (AEs) include hypoglycemia, hypotension, bradycardia, bronchospasm, and sleep disturbances, with growing concern over potential neurodevelopmental effects [11–14]. However, most existing studies are limited by small sample sizes, retrospective designs, and single-center data, leading to incomplete risk characterization and potential reporting bias. To address these limitations, real-world pharmacovigilance studies utilizing large post-marketing surveillance databases have gained importance in rare disease research. The U.S. FDA Adverse Event Reporting System (FAERS) is a global spontaneous reporting database widely used for signal detection and drug safety monitoring [15, 16].

In this context, we conducted a comprehensive pharmacovigilance analysis of FAERS reports related to propranolol use in IH between Q1 2015 and Q4 2024. Multiple disproportionality analysis algorithms—including Reporting Odds Ratio (ROR), Proportional Reporting Ratio (PRR), Bayesian Confidence Propagation Neural Network (BCPNN), and Multi-item Gamma Poisson Shrinker (MGPS)—were applied to identify organ system–level AE signals. This study aims to generate real-world safety evidence for propranolol in the treatment of IH, inform clinical risk management, and support potential updates to drug labeling in pediatric rare disease therapeutics.

## Materials and methods

**Fig. 1:**
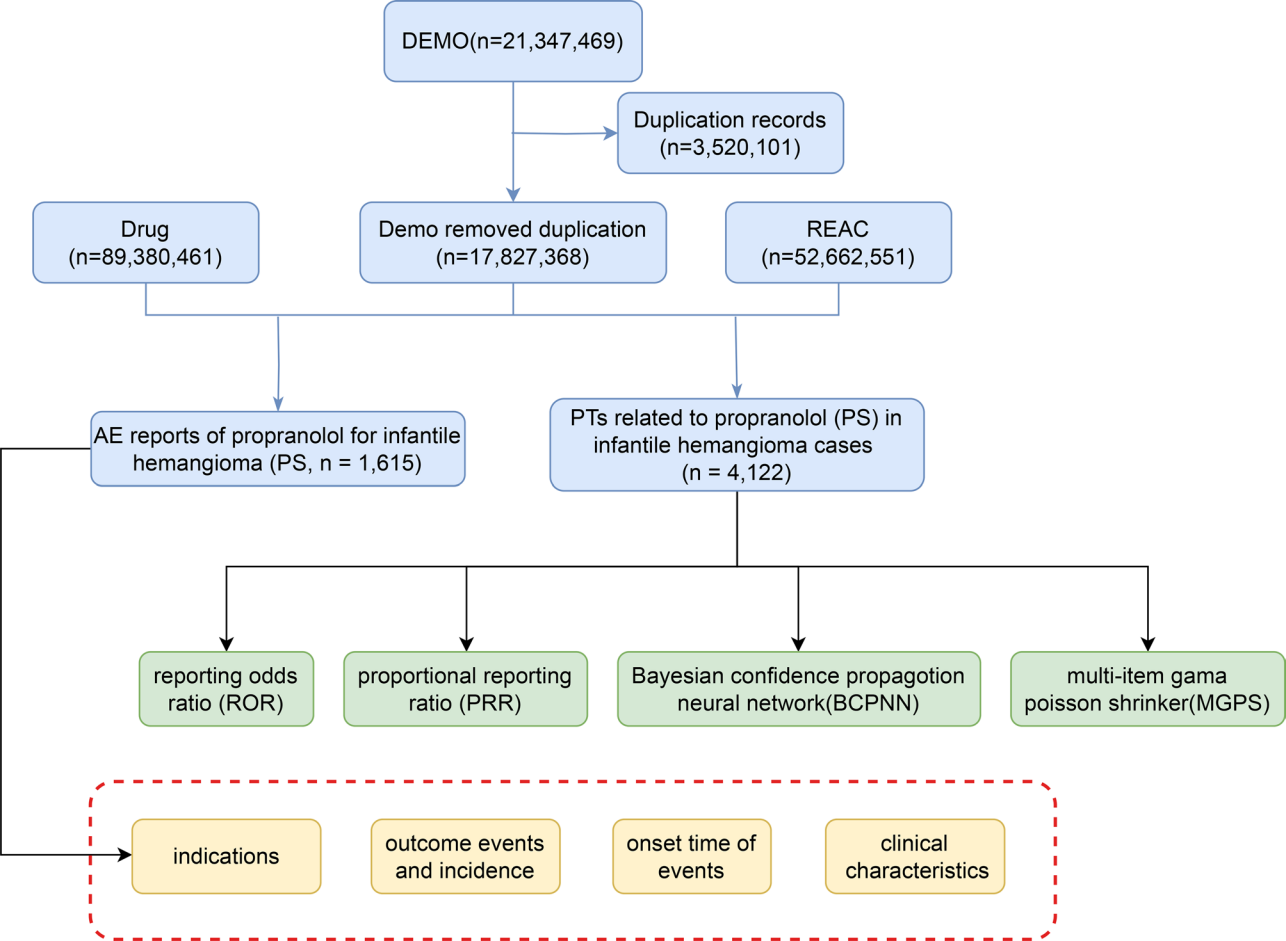
The flow diagram of selecting propranolol-related adverse event reports for infantile hemangioma from the FAERS database

To identify adverse events (AEs) associated with propranolol in the treatment of IH in infants, we conducted a systematic analysis of reports from the FAERS database using four signal detection algorithms. The overall study workflow is illustrated in Fig. 1.

### Data source

This study was based on data from FAERS, a database primarily used for monitoring the safety of drugs and biological products [17]. FAERS is updated quarterly and contains detailed information on individual case safety reports, including drug data, adverse events, clinical outcomes, sources of reports, treatment duration, and indications [18]. We extracted relevant data from Q1 2015 to Q4 2024, and all data processing and analyses were conducted using R software (version 4.4.1).

### Data extraction and analysis

To ensure data accuracy, raw reports from the FAERS database were preprocessed to identify and eliminate duplicates, thereby standardizing the dataset. Both the adverse drug reaction (ADR) terms from FAERS and those used in this study were coded according to the Preferred Terms (PTs) in the Medical Dictionary for Regulatory Activities (MedDRA). Further classification of ADRs was conducted based on the System Organ Class (SOC) categories defined in MedDRA version 23.0. Reports in which propranolol was designated as the primary suspect drug (PSD) were included, and data processing was performed using Microsoft Excel. To minimize indication bias (i.e., misclassification of the drug’s indication as an adverse event), PTs related to the therapeutic indication of propranolol were excluded from the analysis. The extracted parameters included patient sex, age at the time of AE occurrence, body weight, report year, country, type of reporter, outcomes, route of administration, indications, and concomitant medications. Subsequently, we screened for AE reports specifically associated with propranolol use in the treatment of IH in infants [19]. To minimize potential reporting bias and ensure data reliability, the analysis was restricted to adverse event reports submitted by healthcare professionals (specifically pharmacists, physicians, and other health professionals, as categorized in Table 1).

**Table 1:** Basic information on AEs related to propranolol from the FAERS database

Variable	Total(n,%)
**Year**	
2015	34(2.11)
2016	46(2.85)
2017	52(3.22)
2018	104(6.44)
2019	175(10.84)
2020	45(2.79)
2021	35(2.17)
2022	754(46.69)
2023	24(1.49)
2024	346(21.42)
**Sex**	
Female	876(54.24)
Male	316(19.57)
Unknown	423(26.19)
**Age (median (Q1, Q3))**	0.25(0.08,0.75)
**Weight (median (Q1, Q3))**	7.26(5.67,9.07)
**Reporter**	
Pharmacist	988(61.18)
Physician	404(25.02)
Other health-professional	223(13.81)
**Reported countries**	
United States	1146(70.96)
Japan	236(14.61)
Other	233(14.43)
**Route**	
Oral	988(61.18)
Other	627(38.82)
**Outcomes**	
Other serious	285(62.23)
Hospitalization	145(31.66)
Life threatening	14(3.06)
Death	11(2.40)
Disability	3(0.66)
**Time to onset (median(Q1, Q3))**	10.00(1.00,82.00)

Four statistical algorithms were employed for signal detection in this study: Reporting Odds Ratio (ROR) [20], Proportional Reporting Ratio (PRR) [21], Bayesian Confidence Propagation Neural Network (BCPNN) [22], and Multi-item Gamma Poisson Shrinker (MGPS) [23]. ROR, a traditional disproportionality method, enables signal detection even in low-frequency reports and can adjust for certain biases, making it suitable for identifying potential drug–adverse event associations [20, 24]. PRR is comparatively more specific in detecting true signals. BCPNN offers greater sensitivity to low-frequency events, which is advantageous for identifying rare but potentially important adverse reactions [25, 26]. MGPS is also effective in detecting rare-event signals by modeling variance and accounting for multiple comparisons. Additionally, chi-square (χ^2^) tests were applied to assess differences in reporting ratios between groups, and a prioritization system was used to rank signal importance based on magnitude and statistical strength [27].

Frequentist methods (ROR and PRR) are highly sensitive and computationally simple, making them effective for early signal detection, though they may yield higher false-positive rates when event counts are low. In contrast, Bayesian methods (BCPNN and MGPS) employ information shrinkage to adjust for sampling variability, making them more conservative and stable, especially for rare events or small sample sizes. By integrating these methods, we aimed to balance sensitivity and specificity, thereby enhancing the robustness of the detected signals. Corroboration from multiple methodological perspectives was applied to minimize the false-positive rate. Flexible adjustment of algorithmic thresholds and variance estimates further improved sensitivity to rare but clinically relevant adverse events. All computations were based on standard 2 × 2 contingency tables (Supplementary Table 1), with corresponding formulas and signal criteria detailed in Supplementary Table 2. Statistical analyses were conducted using Microsoft Excel 2021 [28, 29].

### Signal selection and classification

PTs with at least three reported cases were selected for signal detection. Subsequently, all signals were standardized, categorized, and mapped using the PTs and SOCs defined by the Medical Dictionary for Regulatory Activities (MedDRA), enabling a structured analysis of adverse event signals within specific SOC categories [24, 30].

## Results

### Basic information on AEs associated with propranolol treatment of IH

**Fig. 2:**
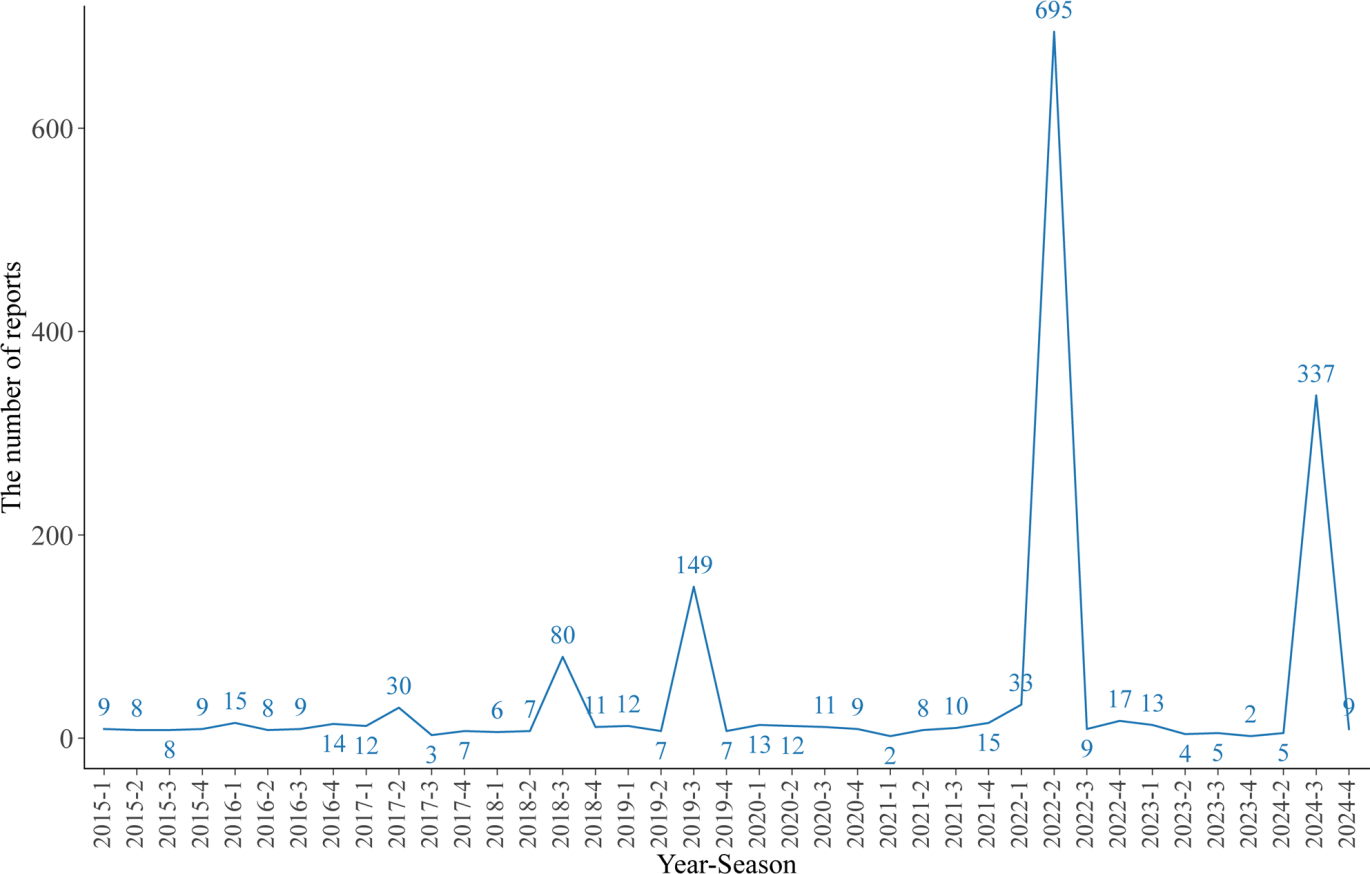
Temporal distribution of adverse event reports associated with propranolol for infantile hemangioma (2015 Q1–2024 Q4)

Between Q1 2015 and Q4 2024, a total of 1615 AE reports were identified in which propranolol was designated as the primary suspect drug for the indication of IH (Table 1). The annual distribution of propranolol-related AEs is illustrated in Fig. 2. The highest numbers of reports were recorded in Q2 2022 (*n* = 695), followed by Q3 2024 (*n* = 337) and Q3 2019 (*n* = 149).

The line graph illustrates the fluctuation in report volume over the ten-year study period. The X-axis represents the timeline by year and quarter, and the Y-axis indicates the total number of adverse event reports submitted to FAERS. The trend highlights notable peaks in reporting frequency, particularly in 2022 Q2 (*n* = 695) and 2024 Q3 (*n* = 337).

In terms of sex distribution, female patients significantly outnumbered males (54.24% vs. 19.57%). The median age of affected individuals was 0.25 years (approximately 3 months), with an interquartile range (IQR) of 0.08–0.75 years. Regarding the timing of AE onset, the median time to onset (TTO) was 10 days, with an IQR of 1–82 days. Most reports were submitted by pharmacists (61.18%), followed by physicians (25.02%) and other healthcare professionals (13.81%). Geographically, the majority of reports originated from the United States (70.96%) and Japan (14.61%), accounting for a combined total of 85.57%. In terms of clinical outcomes, apart from the “other serious” category (62.23%), hospitalization was the most commonly reported outcome (31.66%), followed by life-threatening events (3.06%) and death (2.40%). Detailed information is provided in Table 1.

### Signal detection analysis of propranolol

**Fig. 3:**
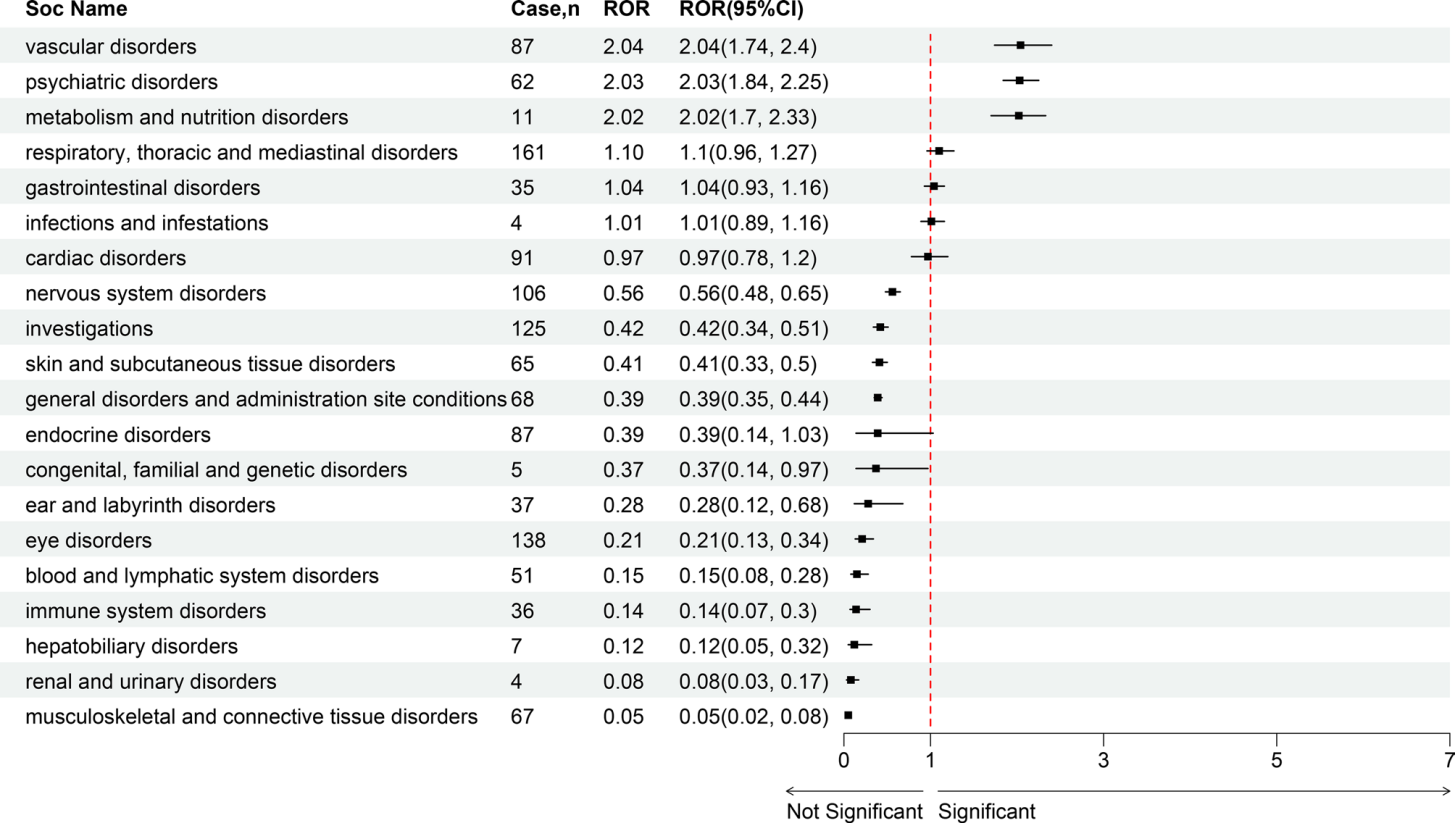
Forest plot of disproportionality analysis (ROR) for SOC-level adverse events associated with propranolol use in infantile hemangioma

The analysis of AE reports related to propranolol use for IH identified adverse events spanning 20 SOCs. The three most prominent SOCs were vascular disorders (*n* = 87, ROR = 2.04), psychiatric disorders (*n* = 62, ROR = 2.03), and metabolism and nutrition disorders (*n* = 11, ROR = 2.02) (Fig. 3).

At the PT level, a total of 75 potential signals were identified using the four signal detection algorithms. Based on the ROR method, PTs were ranked by signal strength according to the 95% confidence interval, with infantile spitting up showing the strongest signal (*n* = 36, ROR = 1361.33).The ten most frequently reported PTs were sleep disorder (*n* = 149, ROR = 32.67), peripheral coldness (*n* = 87, ROR = 108.73), hypoglycaemia (*n* = 62, ROR = 22.95), ear infection (*n* = 52, ROR = 28.95), insomnia (*n* = 50, ROR = 3.01), bradycardia (*n* = 48, ROR = 14.95), decreased appetite (*n* = 47, ROR = 3.02), sleep terror (*n* = 40, ROR = 146.93), infantile spitting up, and irritability (*n* = 34, ROR = 9.46) (Table 2; full list in Supplementary Table 3).

**Table 2:** Top 10 PT-Level adverse event signals identified for propranolol in FAERS

System organ class	PTs		Case Reports	ROR (95% CI)	PRR (95% CI)	χ2	IC (IC025)	EBGM (EBGM05)
Psychiatric disorders	sleep disorder	149		32.67(27.73, 38.49)	31.45(26.89, 36.79)	4383.55	4.97(4.73)	31.35(27.33)
Vascular disorders	peripheral coldness	87		108.73(87.81, 134.63)	106.3(85.68, 131.88)	8978.65	6.72(6.41)	105.16(87.94)
Metabolism and nutrition disorders	hypoglycaemia	62		22.95(17.85, 29.51)	22.6(17.52, 29.16)	1278	4.5(4.14)	22.55(18.28)
Infections and infestations	ear infection	52		28.95(22.01, 38.07)	28.57(21.71, 37.59)	1380.05	4.83(4.44)	28.49(22.65)
Psychiatric disorders	insomnia	50		3.01(2.27, 3.97)	2.98(2.26, 3.92)	66.07	1.58(1.18)	2.98(2.36)
Cardiac disorders	bradycardia	48		14.95(11.24, 19.88)	14.78(11.23, 19.45)	616.05	3.88(3.48)	14.75(11.63)
Metabolism and nutrition disorders	decreased appetite	47		3.02(2.26, 4.02)	2.99(2.27, 3.93)	62.53	1.58(1.17)	2.99(2.35)
Psychiatric disorders	sleep terror	40		146.93(107.35, 201.09)	145.41(106.27, 198.97)	5652.56	7.16(6.72)	143.28(110.19)
Gastrointestinal disorders	infantile spitting up	36		1361.33(959.25, 1931.95)	1348.65(947.72, 1919.19)	42563.63	10.21(9.71)	1184.19(883.51)

### Distribution of PTs across different SOCs

**Fig. 4:**
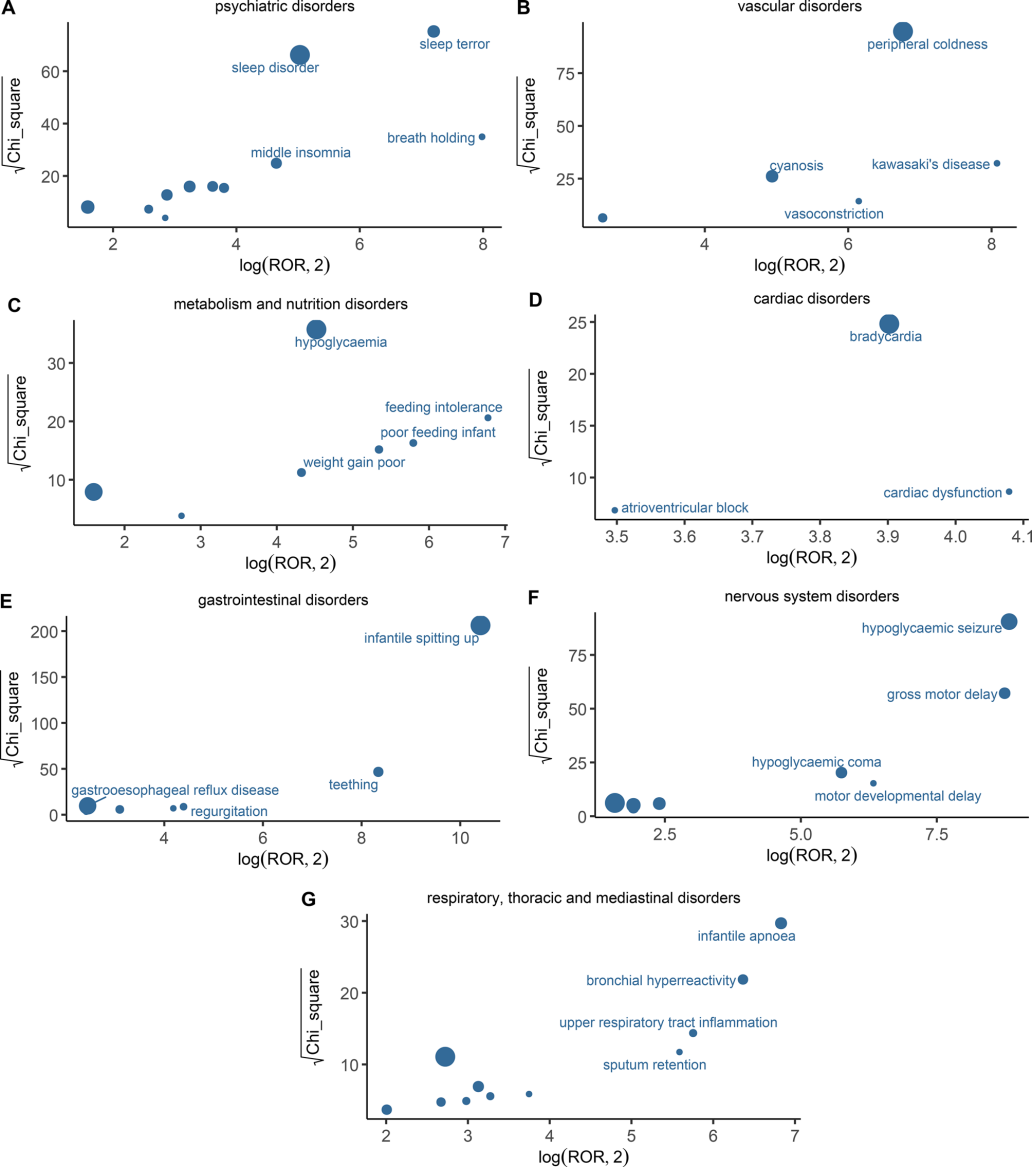
Disproportionality signal visualization of preferred terms (PTs) across seven key system organ classes (SOCs) bubble plots (panels **A**–**G**) depict the strength and statistical robustness of adverse event signals associated with propranolol across seven SOCs. The x-axis shows the base-2 logarithm of the reporting odds ratio (log₂ROR), indicating signal magnitude, and the y-axis shows the square root of the chi-square statistic (√χ^2^), reflecting statistical significance. Bubble size is proportional to the number of reported cases for each PT. PTs located in the upper-right area represent signals with stronger disproportionality and greater statistical reliability. Panels correspond to: (**A**) psychiatric disorders, (**B**) vascular disorders, (**C**) metabolism and nutrition disorders, (**D**) cardiac disorders, (**E**) gastrointestinal disorders, (**F**) nervous system disorders, (**G**) respiratory, thoracic and mediastinal disorders

We analyzed the distribution patterns of propranolol-associated AE signals across seven key SOCs, revealing variable signal intensities and clustering characteristics (Fig. 4).

Notably, several PTs in these SOCs were located in the upper-right quadrant of the log₂ROR versus χ^2^ plot, indicating both a strong association with propranolol exposure and robust statistical significance. The most prominent PTs within each SOC were as follows: sleep terror in psychiatric disorders, peripheral coldness in vascular disorders, hypoglycaemia in metabolism and nutrition disorders, bradycardia in cardiac disorders, infantile spitting up in gastrointestinal disorders, hypoglycaemic seizure in nervous system disorders, and infantile apnoea in respiratory disorders.

These signals, characterized by both high disproportionality and statistical strength, suggest markedly elevated reporting frequencies in the real-world dataset. They warrant heightened clinical vigilance in the infant population and underscore the need for targeted monitoring and individualized risk management strategies.

### Concomitant medication analysis in propranolol-treated IH cases

Among the adverse event reports associated with propranolol treatment for IH, the most frequently reported concomitant medications were timolol (9.25%), cholecalciferol (5.20%), prednisolone (4.05%), and furosemide (4.05%) (Table 3).

**Table 3:** Top six concomitant medications in the treatment of IH with propranolol

	Propranolol	(n, %)
Indication	Infantile hemangioma	173, (100)
Concomitant medication	Timolol	16, (9.25)
	Cholecalciferol	9, (5.20)
	Prednisolone	7, (4.05)
	Furosemide	7, (4.05)
	Incremin	6, (3.47)
	Ranitidine	6, (3.47)
	Spironolactone	6, (3.47)

## Discussion

This study systematically evaluated the distribution characteristics and signal strength of propranolol-associated adverse events (AEs) in the treatment of IH using data from FAERS and multiple signal detection methods, including ROR, PRR, IC, and EBGM. Supported by large-scale real-world data, the results revealed prominent AE signals across several SOCs, particularly the cardiac, psychiatric, and metabolism and nutrition disorders, suggesting that propranolol may pose broad systemic safety risks in the infant population. Compared with conventional clinical trials, this study leverages real-world pharmacovigilance data to fill the evidence gap regarding drug safety in infants. Due to ethical and practical constraints, this population is often underrepresented in randomized controlled trials, and systematic risk warnings are frequently lacking in drug labeling. The large-scale retrospective data provided by the FAERS database facilitates early detection of potential adverse events and enhances risk signal surveillance in this vulnerable group.

Our findings are largely consistent with the safety profile established in pivotal randomized controlled trials [10] and systematic reviews [11], particularly regarding the risks of hypoglycemia and bronchial hyperreactivity. However, our real-world analysis revealed a more pronounced signal for severe sleep disturbances (e.g., “sleep terrors”) and neuropsychiatric events compared to clinical trials, likely reflecting the broader and unselected patient population in routine practice [31–33]. These results support current clinical guidelines that emphasize cardiovascular and metabolic monitoring but suggest that future guidelines [5] could be strengthened by including more specific surveillance protocols for neuropsychiatric and sleep-related adverse events.

Although propranolol has been widely established as a first-line therapy for IH with its efficacy validated in numerous studies, adverse events may still impact treatment adherence and therapeutic outcomes. It has been reported that approximately 2.1% of patients discontinued propranolol prematurely due to intolerable AEs, and a subset of these patients experienced lesion rebound following drug withdrawal, necessitating re-initiation of therapy [31].

In summary, although propranolol demonstrates well-established efficacy in the treatment of IH, clinicians should remain vigilant regarding its potential to induce systemic adverse events in infants—particularly those risks that are insufficiently addressed in current drug labeling or are specific to this vulnerable population. The following sections provide a system-based analysis of the major safety signals identified and offer corresponding risk mitigation strategies.

### Cardiovascular and vascular systems: established mechanisms highlight the importance of rhythm monitoring

Propranolol, a classical non-selective β-adrenergic receptor blocker, has long been utilized in the management of adult cardiovascular conditions such as hypertension, arrhythmias, and angina pectoris. Its therapeutic efficacy primarily stems from β₁-receptor antagonism, which results in reduced heart rate, delayed atrioventricular conduction, and diminished myocardial contractility—effects that underlie its antiarrhythmic and cardioprotective properties [34]. The drug label explicitly lists adverse reactions including bradycardia, hypotension, and impaired cardiac function, with recommendations for close monitoring during treatment initiation or in patients with underlying cardiovascular disorders.

In this study, disproportionality analysis using the FAERS database revealed significant associations between propranolol and cardiac rhythm or functional abnormalities, such as bradycardia (*n* = 48, ROR = 14.95), atrioventricular block (*n* = 5, ROR = 11.29), and cardiac dysfunction (*n* = 5, ROR = 16.9)—findings that are pharmacologically consistent with β₁-receptor blockade [35]. These risks are particularly concerning in infants, whose cardiac structures and conduction systems are still immature, and whose autonomic regulation is underdeveloped, increasing their vulnerability to adverse hemodynamic effects and risk of rapid decompensation. Vascular-related adverse event signals were also prominent. Notably, peripheral coldness (*n* = 87, ROR = 108.73), vasoconstriction (*n* = 3, ROR = 70.88), and cyanosis (*n* = 24, ROR = 30.66) were identified, likely due to β₂-receptor–mediated vasoconstriction and reduced thermogenesis secondary to inhibition of brown adipose tissue activity [36]. Given the immature thermoregulatory capacity and low basal metabolic rate in infants, these findings raise concern for compromised peripheral perfusion and thermal instability.

Based on these safety signals, routine monitoring of heart rate, blood pressure, and electrocardiographic parameters is recommended throughout propranolol therapy, particularly during initiation and dose escalation. Clinicians should be vigilant for early signs of hypoperfusion, including peripheral coldness and cyanosis, which may indicate developing circulatory compromise. In high-risk populations—such as preterm infants, those with low birth weight, or concurrent infections—enhanced thermal support and individualized cardiovascular risk stratification are strongly advised to mitigate perfusion-related complications and optimize overall treatment safety.

### Respiratory system adverse events: vigilance for bronchial hyperreactivity

Propranolol-induced blockade of β₂-adrenergic receptors can result in bronchial smooth muscle constriction, increased airway resistance, and impaired mucociliary clearance [37], which pose particular risks in infants due to their immature pulmonary architecture and limited respiratory reserve. Signal detection from the FAERS database revealed markedly elevated disproportionality for severe respiratory events, including infantile apnoea (*n* = 8, ROR = 113.72) and bronchial hyperreactivity (*n* = 6, ROR = 82.35), indicating a substantial real-world safety concern. Additionally, wheezing (*n* = 26, ROR = 6.6) was reported more frequently, reflecting both its higher clinical prevalence and potential for early respiratory compromise. These respiratory events may manifest insidiously, with nonspecific signs such as diminished crying, cyanosis, nasal flaring, or feeding difficulties—symptoms that can be easily misattributed to infection or gastrointestinal disorders, potentially delaying appropriate intervention. Recent studies have indicated that infants with a prior history of apnea or neonatal pneumonia may be at increased risk of developing bronchial hyperreactivity following the initiation of non-selective β-blocker therapy [38], underscoring the need for targeted risk assessment in this subgroup.

Therefore, before initiating propranolol in the treatment of IH, a thorough evaluation of the infant’s respiratory history and baseline pulmonary function is essential. In high-risk infants, propranolol should be prescribed with caution, and continuous respiratory monitoring should be maintained throughout treatment. Additionally, caregivers should be educated to identify abnormal breathing patterns, enabling timely clinical response in the event of adverse respiratory symptoms.

### Neurologic and psychiatric AEs: prominent signals of sleep and behavioral disturbances raise concerns for neurodevelopmental interference in infants

FAERS-based analysis revealed a significant association between propranolol use for IH and various neurologic and psychiatric AEs. Its lipophilic nature allows it to cross the blood–brain barrier and potentially interfere with circadian rhythms, neurotransmitter balance, and neurodevelopment via central β-adrenergic antagonism [33, 39]. At the psychiatric level, sleep disorder (*n* = 149, ROR = 32.67) was the most frequently reported AE, followed by other sleep-related signals such as insomnia (*n* = 50, ROR = 3.01), sleep terror (*n* = 40, ROR = 146.93), and middle insomnia (*n* = 27, ROR = 25.07), all of which showed strong positive signals. Beyond sleep disturbances, emotional and behavioral abnormalities such as agitation (*n* = 30, ROR = 7.33) and irritability (*n* = 34, ROR = 9.46) were also frequently reported, suggesting that propranolol may induce a range of psychiatric manifestations by disrupting the balance of central neurotransmitters. On the neurologic side, hypoglycaemic seizure (*n* = 19, ROR = 455.19) and hypoglycaemic coma (*n* = 8, ROR = 53.68) exhibited exceptionally strong signals, implicating propranolol in exacerbating hypoglycaemia-induced neurotoxicity and posing risks to infant neurodevelopment. Supporting these signals, a cohort study reported more nighttime awakenings and lower sleep efficiency in infants treated with propranolol [32]. In a retrospective analysis of 1260 patients, 65.4% of treatment discontinuations due to AEs were related to sleep disturbances, making it the leading cause of withdrawal [31]. Preclinical studies suggest that central β-adrenergic signaling is vital for synaptic plasticity and neurocognitive development, and long-term blockade may impair learning and memory [40]. Although some prospective clinical studies found no significant neurodevelopmental impact [13], the vulnerability of the infant CNS and individual differences underscore the need for close monitoring.

In summary, propranolol may interfere with CNS signaling and contribute to sleep, behavioral, and developmental disturbances. Baseline neurologic assessment, ongoing observation of sleep/emotional states, and caregiver education are recommended. If serious AEs occur, clinical reassessment and treatment adjustment should be promptly considered.

### Significant signals of metabolic disorders: hypoglycaemia and its CNS sequelae require vigilant monitoring

In the metabolic disorders system, hypoglycaemia (*n* = 62, ROR = 22.95) emerged as the most frequently reported adverse event, exhibiting a notably strong signal. Additional events such as feeding intolerance (*n* = 4, ROR = 109.44) and poor weight gain (*n* = 6, ROR = 40.57) also demonstrated elevated disproportionality, indicating broader nutritional and metabolic vulnerabilities in infants treated with propranolol. Mechanistically, propranolol inhibits glycogenolysis, gluconeogenesis, and lipolysis through β-adrenergic blockade, thereby impairing endogenous glucose production. It also suppresses adrenergic warning signs—such as tremor and sweating—by attenuating sympathetic responses [41], making asymptomatic hypoglycaemia more likely and harder to detect, especially in nonverbal populations. This metabolic disturbance may predispose infants to serious central nervous system injury. Strong signals were observed for hypoglycaemic seizure (*n* = 19, ROR = 455.19) and hypoglycaemic coma (*n* = 8, ROR = 53.68), suggesting that hypoglycaemia is not only prevalent but also a critical precipitant of neurotoxicity. Given infants’ limited metabolic reserves and ongoing neural development, even brief episodes of glucose deprivation can result in seizures, altered consciousness, and potentially irreversible neurodevelopmental damage [42].

While current labeling acknowledges the risk of hypoglycaemia, it lacks detailed guidance regarding CNS consequences. Therefore, clinicians should assess nutritional and metabolic baselines—including birth weight, feeding ability, and glucose levels—prior to treatment. Propranolol should be administered after feeding, and prolonged fasting, particularly overnight, should be avoided. During therapy, close blood glucose monitoring is essential, and caregivers must be educated to recognize early signs of both hypoglycaemia and CNS involvement. A multilayered prevention strategy is key to minimizing metabolism-related neurological complications.

### General disorders and drug withdrawal: vital sign abnormalities indicate sympathetic fluctuation risk

FAERS data revealed notable signals of vital sign abnormalities in the General disorders and administration site conditions and Investigations SOCs. Among them, “heart rate decreased” (*n* = 12, ROR = 4.99) and “blood pressure decreased” (*n* = 15, ROR = 3.76) were the most frequently reported, suggesting that propranolol may induce systemic physiological suppression in certain infants. In addition, “drug withdrawal syndrome neonatal” (*n* = 9, ROR = 7.27) also showed a strong signal, indicating that abrupt discontinuation after prolonged or high-dose administration may lead to rebound sympathetic activity, clinically manifested as instability in vital signs such as heart rate and blood pressure. This highlights the need for careful withdrawal management in high-risk infants. The underlying mechanisms of these adverse events may relate to propranolol’s broad suppression of sympathetic activity, reduced basal metabolic rate, and impaired thermogenesis. As thermoregulation and autonomic reflexes are immature in infants, they are more sensitive to the effects of β-blockers and more prone to excessive physiological suppression.

Therefore, for infants receiving long-term propranolol therapy—especially those with underlying cardiovascular conditions or neurodevelopmental disorders—individualized risk assessment should be conducted [43]. Tapering regimens should be carefully planned to avoid abrupt discontinuation and the resulting adverse physiological stress responses [44]. During withdrawal, close monitoring of vital signs such as heart rate and blood pressure is recommended to ensure a smooth and safe discontinuation process.

### Adverse events in other systems: multi-system signals require mechanism-based and clinical context interpretation

In addition to cardiovascular, respiratory, neuropsychiatric, metabolic, and general disorders, this study identified several adverse event (AE) signals involving the gastrointestinal and dermatological systems. Although the reporting frequency of these events was relatively low, some were supported by well-established pharmacological mechanisms. Others may have been influenced by comorbid conditions or age-specific developmental characteristics in infants, requiring careful interpretation of drug causality in the context of both mechanism and clinical background.

Among gastrointestinal AEs, “gastrooesophageal reflux disease” (*n* = 26, ROR = 5.45) exhibited a notably strong signal. This association is pharmacologically plausible, as propranolol may reduce lower esophageal sphincter tone through β₂-receptor blockade, thereby predisposing to reflux symptoms [45]. During treatment, close monitoring for feeding intolerance, dysphagia, and reflux manifestations is warranted. Strategies such as administering propranolol after meals or combining it with prokinetic agents may alleviate gastrointestinal discomfort and help prevent secondary complications such as aspiration pneumonia. In cases of persistent gastrointestinal symptoms, dose reduction or individualized treatment adjustment should be considered. Notably, the PT “infantile spitting up” exhibited an exceptionally high ROR (1361.33). This extreme disproportionality warrants cautious interpretation, as it is primarily an artifact of the demographic discrepancy between the target cohort and the background reference population. Because our study cohort exclusively comprises infants, whereas the overall FAERS database is predominantly composed of adult records, pediatric-specific terms are inherently underrepresented in the background data. Consequently, calculating disproportionality for an age-specific event against an all-age reference mathematically inflates the signal magnitude. Therefore, this finding more likely reflects age-related physiological norms and inherent reporting bias rather than a genuine, severe toxicological effect of propranolol.

In the skin and subcutaneous tissue disorders category, notable signals were identified for skin discolouration (*n* = 13, ROR = 4.28) and diaper dermatitis (*n* = 7, ROR = 149.21). The former may reflect propranolol-induced peripheral vasoconstriction, necessitating clinical vigilance for perfusion changes. In contrast, the signal for diaper dermatitis, despite its high statistical strength, requires cautious interpretation. This signal is heavily confounded by the high baseline prevalence of diaper dermatitis in the infant population and likely represents age-related background noise rather than a specific drug-induced adverse event. Clinicians should remain vigilant for skin color changes and local inflammatory signs during therapy, with appropriate skin care and perfusion assessment to enhance safety.

Regarding the frequent reporting of “ear infection,” we consider this signal to be largely coincidental, reflecting the high baseline prevalence of otitis media in the infant population rather than a direct drug toxicity. However, it is worth noting that non-selective β-blockers can induce nasal mucosal congestion, which may theoretically predispose infants to eustachian tube dysfunction and secondary infections. Therefore, while this is likely a background event, clinicians should remain vigilant in distinguishing between drug-induced respiratory symptoms and common pediatric infections to avoid unnecessary discontinuation of therapy.

### Systematic consideration of alternative therapies: balancing safety enhancement and efficacy trade-offs

This study identified significant adverse event signals across multiple organ systems—including neurologic, metabolic, respiratory, cardiovascular, and gastrointestinal—associated with propranolol use for IH. These risks are largely attributable to its pharmacologic profile as a non-selective β-adrenergic receptor blocker, which inhibits both β₁ and β₂ receptors.

In infants at elevated risk—such as those born prematurely, with low birth weight, prior hypoglycaemia, or underlying neurologic or respiratory vulnerabilities—a β₁-selective blocker with lower central nervous system (CNS) penetration, such as atenolol, may be considered. Atenolol is more hydrophilic and less likely to cross the blood–brain barrier, exhibiting weaker β₂ blockade and potentially offering greater safety in terms of neurotoxicity, respiratory depression, and metabolic disturbances. However, clinical data suggest that β₁-selective blockers may be less effective than propranolol in terms of lesion regression speed and complete response rate [46]. This may reflect the critical role of β₂-receptor pathways in regulating angiogenesis and lesion proliferation, for which non-selective β-blockade provides broader therapeutic inhibition.

Therefore, the choice of β-blocker for IH should be guided by a tailored risk–benefit assessment. For infants with high-risk profiles or intolerance to propranolol, β₁-selective agents may offer a safer alternative. Conversely, in cases involving rapidly growing lesions or high-risk anatomical locations, propranolol may remain the preferred option. Further high-quality prospective studies are needed to optimize individualized β-blocker strategies in IH management.

### Limitations

Although this study systematically evaluates the AE profile of propranolol in the treatment of IH using the FAERS database and identified several clinically relevant safety signals, some limitations should be acknowledged. First, as a spontaneous reporting system, FAERS is subject to inherent biases, including underreporting, duplicate entries, and inconsistent data quality. Underreporting implies that calculated RORs may not represent true incidence. To enhance data reliability, the analysis was restricted to reports submitted by healthcare professionals—comprising physicians, pharmacists, and other health professionals (Table 1). However, even with this restriction, the possibility of residual duplicate records or incomplete clinical information cannot be entirely ruled out. Second, both reporting bias and indication bias may confound the interpretation of drug-event associations, particularly in pediatric populations. Third, the lack of key clinical variables such as dosage, treatment duration, comorbidities, and detailed information on concomitant therapies limits further exploration of dose-response relationships and risk stratification. Specifically, although we identified the presence of concomitant medications (e.g., timolol, cholecalciferol) in Table 3, this analysis did not statistically adjust for their potential confounding effects on adverse event signals. The heterogeneous reporting of drug administration timing and dosages in the FAERS database makes it difficult to distinguish whether an observed event was induced solely by propranolol, a concomitant drug, or a synergistic interaction. Furthermore, the geographic distribution of reports in this study was highly skewed, with the United States (70.96%) and Japan (14.61%) accounting for the vast majority of cases. This disproportionate representation may introduce geographic and systemic biases related to regional reporting practices, healthcare policies, or genetic differences. Consequently, the safety signals identified in this analysis may not be fully generalizable to populations in other regions, particularly developing countries or those with different ethnic demographics. Finally, signal detection is correlational by nature and cannot establish causality between propranolol exposure and observed AEs.

Therefore, while this study provides preliminary signal-level evidence with potential alerting value, further validation through prospective cohort studies, pharmacokinetic–pharmacodynamic (PK–PD) modeling, and mechanistic research is warranted to confirm causality and strengthen clinical interpretability. Despite these limitations, FAERS-based pharmacovigilance remains a vital component of real-world evidence generation. Especially for high-risk populations such as infants, it plays a critical role in the early identification of safety concerns, refinement of post-marketing surveillance, and support for lifecycle-based drug safety strategies.

## Conclusions

This pharmacovigilance study analyzed FAERS data on propranolol use in the treatment of IH and identified potential safety signals involving multiple organ systems, including cardiovascular, respiratory, neurologic, metabolic, and psychiatric domains. These findings necessitate the need for heightened clinical vigilance and underscore the importance of individualized treatment planning in this vulnerable population. To mitigate these risks in clinical practice, we propose the following specific monitoring protocols: (1) Routine monitoring of heart rate and blood pressure is essential, particularly during treatment initiation and dose escalation, to detect early signs of bradycardia or hypotension; (2) Caregivers should be educated on the risks of hypoglycemia and instructed to ensure frequent feeding, especially during periods of intercurrent illness or reduced oral intake; (3) Given the strong signals for sleep disorders and neuropsychiatric events, clinicians should actively inquire about sleep patterns and behavioral changes at each follow-up visit, which may warrant dose modification or a switch to hydrophilic beta-blockers.

By providing real-world evidence on the systemic safety risks of propranolol in infants, this study offers valuable insights to inform clinical decision-making and support regulatory risk assessment. However, to address the inherent limitations of spontaneous reporting and the lack of granular clinical variables, future research should leverage electronic medical record (EMR) databases or prospective cohort studies. Such approaches will enable precise evaluation of dosage regimens, treatment duration, and comorbidities, facilitating robust risk stratification and the establishment of definitive causal relationships. The results may serve as a reference for refining β-blocker safety labeling, guiding therapeutic strategies, and advancing proactive risk mitigation in pediatric pharmacotherapy.

## Electronic supplementary material

Below is the link to the electronic supplementary material.

Supplementary Material 1

Supplementary Material 2

Supplementary Material 3

## Data Availability

The datasets analyzed during the current study are publicly available from the FDA Adverse Event Reporting System (FAERS): https://www.fda.gov.
