# Supplementary material for: Orphan drug propranolol for infantile hemangioma: ten-year real-world safety data from the FAERS database
Source: Orphanet J Rare Dis. 2026 Apr 1;21:187. doi: 10.1186/s13023-026-04331-4 (PMC13154883; doi:10.1186/s13023-026-04331-4)
Supplement: Supplementary file 1 — Supplementary Material 1 [file 13023_2026_4331_MOESM1_ESM.docx]

**Table S1.** **Two-by-two contingency table for analyses.**

| Drug | Target adverse events | Non-target adverse events | total |
| --- | --- | --- | --- |
| Propranolol | a | b | a+b |
| Non-propranolol | c | d | c+d |
| Total | a+c | b+d | a+b+c+d |
