# Supplementary material for: Orphan drug propranolol for infantile hemangioma: ten-year real-world safety data from the FAERS database
Source: Orphanet J Rare Dis. 2026 Apr 1;21:187. doi: 10.1186/s13023-026-04331-4 (PMC13154883; doi:10.1186/s13023-026-04331-4)
Supplement: Supplementary file 2 — Supplementary Material 2 [file 13023_2026_4331_MOESM2_ESM.docx]

**Table S2. Principle of dis-proportionality measure and standard of signal detection.**

| **Method** | **Formula** | **Threshold** |
| --- | --- | --- |
| *ROR* | $ROR=\frac{\left( a/c \right)}{\left( b/d \right)}=\frac{ad}{bc}$ | a≥3 and 95% CI  （lower limit）  >1 |
|  | $SE(lnROR)=\sqrt{(\frac{1}{a}+\frac{1}{b}+\frac{1}{c}+\frac{1}{d})}$ |  |
|  | $95\%CI=e^{ln\left( ROR \right)\pm1.96}\sqrt{(\frac{1}{a}+\frac{1}{b}+\frac{1}{c}+\frac{1}{d})}$ |  |
| *PRR* | $PRR=\frac{a/(a+b)}{c/(c+d)}$ | a≥3 and 95% CI  （lower limit）  >1 |
|  | $SE(lnPRR)=\sqrt{\frac{1}{a}-\frac{1}{a+b}+\frac{1}{c}-\frac{1}{c+d}}$ |  |
|  | $95\%CI=e^{ln(PRR)\pm1.96}\sqrt{\frac{1}{a}-\frac{1}{a+b}+\frac{1}{c}-\frac{1}{c+d}}$ |  |
| *BCPNN* | $IC=log_{2}\frac{p(x,y)}{p(x)p(y)}=log_{2}\frac{a(a+b+c+d)}{\left( a+b)(a+c \right)}$ | IC025 > 0 |
|  | $E(IC)=log_{2}\frac{\left( a+\gamma11)(a+b+c+d+\alpha)(a+b+c+d+\beta\right)}{\left( a+b+c+d+\gamma)(a+b+\alpha1)(a+c+\beta1 \right)}$ |  |
|  | $V(IC)=\frac{1}{\left( \ln2 \right)^{2}}\{[\frac{(a+b+c+d)-a+\gamma-\gamma11}{\left( a+\gamma11)(1+a+b+c+d+\gamma\right)}]+[\frac{(a+b+c+d)-(a+b)+\alpha-\alpha1}{\left( a+b+\alpha1)(1+\alpha+b+c+d+\alpha\right)}]+ [\frac{(a+b+c+d)-(a+c)+\beta-\beta1}{\left( a+c+\beta1)(1+a+b+c+d+\beta\right)}]\}$ |  |
|  | $\gamma=\gamma11\frac{\left( a+b+c+d+\alpha)(a+b+c+d+\beta\right)}{\left( a+b+\alpha1)(a+c+\beta1 \right)}$ |  |
|  | $IC-2SD=E(IC)-2\sqrt{V(IC)}$ |  |
| *EBGM* | $EBGM=\frac{a(a+b+c+d)}{\left( a+c)(a+b \right)}$ | EBGM05 > 2 |
|  | $95\%CI=e^{ln\left( EBGM \right)\pm1.96\sqrt{(\frac{1}{a}+\frac{1}{b}+\frac{1}{c}+\frac{1}{d})}}$ |  |
