# Supplementary material for: Orphan drug propranolol for infantile hemangioma: ten-year real-world safety data from the FAERS database
Source: Orphanet J Rare Dis. 2026 Apr 1;21:187. doi: 10.1186/s13023-026-04331-4 (PMC13154883; doi:10.1186/s13023-026-04331-4)
Supplement: Supplementary file 3 — Supplementary Material 3 [file 13023_2026_4331_MOESM3_ESM.doc]

**Table S3. The signal strength of AEs of Propranolol at the PTs level in FAERS database**

|  | **soc_english** | **pt_english** | **Case Reports** | **ROR**  **(95% CI)** | **PRR**  **(95% CI)** | **chisq** | **IC(IC025)** | **EBGM**  **(EBGM05)** |
| --- | --- | --- | --- | --- | --- | --- | --- | --- |
| 1 | respiratory, thoracic and mediastinal disorders | wheezing | 26 | 6.6(4.49, 9.7) | 6.56(4.43, 9.71) | 122.57 | 2.71(2.17) | 6.56(4.75) |
| 2 | respiratory, thoracic and mediastinal disorders | infantile apnoea | 8 | 113.72(56.6, 228.48) | 113.48(56.04, 229.8) | 881.64 | 6.81(5.86) | 112.18(62.57) |
| 3 | respiratory, thoracic and mediastinal disorders | bronchospasm | 7 | 8.74(4.16, 18.35) | 8.72(4.14, 18.36) | 47.83 | 3.12(2.12) | 8.72(4.69) |
| 4 | respiratory, thoracic and mediastinal disorders | bronchial hyperreactivity | 6 | 82.35(36.85, 184.05) | 82.23(36.82, 183.67) | 477.4 | 6.35(5.27) | 81.54(41.61) |
| 5 | respiratory, thoracic and mediastinal disorders | sneezing | 6 | 4.02(1.81, 8.96) | 4.02(1.8, 8.98) | 13.6 | 2.01(0.94) | 4.02(2.05) |
| 6 | respiratory, thoracic and mediastinal disorders | pulmonary congestion | 5 | 6.37(2.65, 15.31) | 6.36(2.63, 15.36) | 22.57 | 2.67(1.51) | 6.35(3.05) |
| 7 | respiratory, thoracic and mediastinal disorders | apnoea | 4 | 9.67(3.63, 25.79) | 9.66(3.63, 25.74) | 31.02 | 3.27(2) | 9.65(4.25) |
| 8 | respiratory, thoracic and mediastinal disorders | respiration abnormal | 4 | 7.89(2.96, 21.04) | 7.88(2.96, 21) | 24.01 | 2.98(1.71) | 7.87(3.47) |
| 9 | respiratory, thoracic and mediastinal disorders | upper respiratory tract inflammation | 4 | 53.96(20.19, 144.24) | 53.9(20.23, 143.61) | 206.55 | 5.74(4.47) | 53.61(23.55) |
| 10 | respiratory, thoracic and mediastinal disorders | respiratory symptom | 3 | 13.43(4.33, 41.69) | 13.42(4.31, 41.83) | 34.44 | 3.74(2.33) | 13.4(5.19) |
| 11 | respiratory, thoracic and mediastinal disorders | sputum retention | 3 | 48.07(15.45, 149.53) | 48.03(15.41, 149.7) | 137.48 | 5.58(4.16) | 47.8(18.5) |
| 12 | psychiatric disorders | sleep disorder | 149 | 32.67(27.73, 38.49) | 31.45(26.89, 36.79) | 4383.55 | 4.97(4.73) | 31.35(27.33) |
| 13 | psychiatric disorders | insomnia | 50 | 3.01(2.27, 3.97) | 2.98(2.26, 3.92) | 66.07 | 1.58(1.18) | 2.98(2.36) |
| 14 | psychiatric disorders | sleep terror | 40 | 146.93(107.35, 201.09) | 145.41(106.27, 198.97) | 5652.56 | 7.16(6.72) | 143.28(110.19) |
| 15 | psychiatric disorders | irritability | 34 | 9.46(6.75, 13.26) | 9.38(6.72, 13.09) | 254.65 | 3.23(2.75) | 9.38(7.07) |
| 16 | psychiatric disorders | agitation | 30 | 7.33(5.12, 10.5) | 7.28(5.12, 10.36) | 162.7 | 2.86(2.35) | 7.28(5.39) |
| 17 | psychiatric disorders | middle insomnia | 27 | 25.07(17.16, 36.62) | 24.9(17.16, 36.14) | 617.92 | 4.63(4.1) | 24.84(18.09) |
| 18 | psychiatric disorders | nightmare | 25 | 12.25(8.26, 18.15) | 12.17(8.22, 18.01) | 256.19 | 3.6(3.05) | 12.16(8.75) |
| 19 | psychiatric disorders | poor quality sleep | 20 | 13.92(8.96, 21.6) | 13.85(9, 21.32) | 238.16 | 3.79(3.17) | 13.83(9.57) |
| 20 | psychiatric disorders | restlessness | 13 | 5.97(3.46, 10.29) | 5.95(3.44, 10.3) | 53.58 | 2.57(1.82) | 5.95(3.77) |
| 21 | psychiatric disorders | breath holding | 5 | 253(104.06, 615.13) | 252.68(104.6, 610.41) | 1221.6 | 7.94(6.77) | 246.29(117.11) |
| 22 | psychiatric disorders | behaviour disorder | 3 | 7.19(2.32, 22.31) | 7.19(2.31, 22.41) | 15.96 | 2.84(1.43) | 7.18(2.78) |
| 23 | general disorders and administration site conditions | disease recurrence | 29 | 8.07(5.6, 11.63) | 8.01(5.52, 11.62) | 178.02 | 3(2.48) | 8.01(5.9) |
| 24 | general disorders and administration site conditions | crying | 14 | 6.83(4.04, 11.54) | 6.81(4.01, 11.56) | 69.32 | 2.77(2.03) | 6.8(4.38) |
| 25 | general disorders and administration site conditions | ulcer | 10 | 10.34(5.56, 19.23) | 10.31(5.51, 19.3) | 84.02 | 3.36(2.51) | 10.3(6.13) |
| 26 | general disorders and administration site conditions | drug withdrawal syndrome neonatal | 9 | 7.27(3.78, 13.98) | 7.25(3.8, 13.84) | 48.5 | 2.86(1.96) | 7.25(4.19) |
| 27 | general disorders and administration site conditions | paradoxical drug reaction | 8 | 31.8(15.87, 63.7) | 31.73(15.98, 63.01) | 237.37 | 4.98(4.04) | 31.63(17.69) |
| 28 | general disorders and administration site conditions | hypothermia | 7 | 11.49(5.47, 24.14) | 11.47(5.45, 24.16) | 66.87 | 3.52(2.52) | 11.46(6.16) |
| 29 | general disorders and administration site conditions | sudden infant death syndrome | 5 | 216.86(89.34, 526.41) | 216.58(89.65, 523.2) | 1049.5 | 7.73(6.56) | 211.87(100.88) |
| 30 | general disorders and administration site conditions | screaming | 4 | 11.94(4.48, 31.85) | 11.93(4.48, 31.79) | 40 | 3.57(2.31) | 11.91(5.24) |
| 31 | general disorders and administration site conditions | ulcer haemorrhage | 3 | 7.85(2.53, 24.35) | 7.84(2.52, 24.44) | 17.89 | 2.97(1.55) | 7.84(3.04) |
| 32 | nervous system disorders | seizure | 29 | 2.99(2.07, 4.31) | 2.97(2.05, 4.31) | 38.07 | 1.57(1.05) | 2.97(2.19) |
| 33 | nervous system disorders | hypoglycaemic seizure | 19 | 455.19(287.03, 721.86) | 452.96(288.59, 710.95) | 8186.14 | 8.76(8.11) | 432.8(294.25) |
| 34 | nervous system disorders | lethargy | 13 | 3.79(2.2, 6.53) | 3.78(2.18, 6.54) | 26.58 | 1.92(1.16) | 3.78(2.4) |
| 35 | nervous system disorders | epilepsy | 10 | 5.27(2.83, 9.8) | 5.26(2.81, 9.85) | 34.46 | 2.39(1.54) | 5.25(3.13) |
| 36 | nervous system disorders | gross motor delay | 8 | 429.74(211.53, 873.05) | 428.85(211.77, 868.45) | 3270.31 | 8.68(7.72) | 410.74(226.98) |
| 37 | nervous system disorders | hypoglycaemic coma | 8 | 53.68(26.77, 107.62) | 53.57(26.98, 106.38) | 410.46 | 5.74(4.79) | 53.28(29.77) |
| 38 | nervous system disorders | hypersomnia | 7 | 3.79(1.8, 7.95) | 3.78(1.79, 7.96) | 14.33 | 1.92(0.92) | 3.78(2.03) |
| 39 | nervous system disorders | motor developmental delay | 3 | 80.69(25.89, 251.49) | 80.63(25.87, 251.31) | 233.99 | 6.32(4.9) | 79.98(30.89) |
| 40 | metabolism and nutrition disorders | hypoglycaemia | 62 | 22.95(17.85, 29.51) | 22.6(17.52, 29.16) | 1278 | 4.5(4.14) | 22.55(18.28) |
| 41 | metabolism and nutrition disorders | decreased appetite | 47 | 3.02(2.26, 4.02) | 2.99(2.27, 3.93) | 62.53 | 1.58(1.17) | 2.99(2.35) |
| 42 | metabolism and nutrition disorders | hypertriglyceridaemia | 7 | 20.03(9.54, 42.09) | 20(9.5, 42.12) | 126.1 | 4.32(3.32) | 19.96(10.73) |
| 43 | metabolism and nutrition disorders | weight gain poor | 6 | 40.57(18.19, 90.52) | 40.51(18.14, 90.48) | 230.27 | 5.33(4.26) | 40.35(20.62) |
| 44 | metabolism and nutrition disorders | poor feeding infant | 5 | 55.45(23.01, 133.64) | 55.38(22.92, 133.78) | 265.49 | 5.78(4.62) | 55.07(26.38) |
| 45 | metabolism and nutrition disorders | feeding intolerance | 4 | 109.44(40.83, 293.35) | 109.33(41.03, 291.31) | 424.57 | 6.76(5.48) | 108.12(47.38) |
| 46 | metabolism and nutrition disorders | appetite disorder | 3 | 6.72(2.17, 20.85) | 6.71(2.15, 20.91) | 14.58 | 2.75(1.33) | 6.71(2.6) |
| 47 | infections and infestations | ear infection | 52 | 28.95(22.01, 38.07) | 28.57(21.71, 37.59) | 1380.05 | 4.83(4.44) | 28.49(22.65) |
| 48 | infections and infestations | respiratory syncytial virus infection | 18 | 32.71(20.57, 52.02) | 32.57(20.35, 52.13) | 548.97 | 5.02(4.37) | 32.46(22.02) |
| 49 | infections and infestations | upper respiratory tract infection | 14 | 4.59(2.72, 7.76) | 4.58(2.7, 7.77) | 39.18 | 2.19(1.46) | 4.58(2.95) |
| 50 | infections and infestations | viral infection | 8 | 3.82(1.91, 7.65) | 3.82(1.92, 7.59) | 16.65 | 1.93(0.99) | 3.82(2.14) |
| 51 | infections and infestations | gastroenteritis viral | 6 | 5.02(2.25, 11.19) | 5.02(2.25, 11.21) | 19.29 | 2.33(1.26) | 5.01(2.57) |
| 52 | infections and infestations | bronchiolitis | 5 | 23.48(9.76, 56.5) | 23.45(9.71, 56.65) | 107.2 | 4.55(3.39) | 23.39(11.22) |
| 53 | infections and infestations | gastroenteritis | 4 | 4.68(1.75, 12.47) | 4.67(1.75, 12.44) | 11.54 | 2.22(0.96) | 4.67(2.06) |
| 54 | gastrointestinal disorders | infantile spitting up | 36 | 1361.33(959.25, 1931.95) | 1348.65(947.72, 1919.19) | 42563.63 | 10.21(9.71) | 1184.19(883.51) |
| 55 | gastrointestinal disorders | gastrooesophageal reflux disease | 26 | 5.45(3.71, 8.02) | 5.42(3.66, 8.02) | 93.81 | 2.44(1.89) | 5.42(3.92) |
| 56 | gastrointestinal disorders | teething | 7 | 324.01(152.48, 688.49) | 323.43(153.57, 681.15) | 2177.44 | 8.29(7.27) | 313.03(166.6) |
| 57 | gastrointestinal disorders | faeces soft | 5 | 8.56(3.56, 20.59) | 8.55(3.54, 20.65) | 33.33 | 3.1(1.94) | 8.55(4.1) |
| 58 | gastrointestinal disorders | regurgitation | 4 | 20.97(7.86, 55.95) | 20.95(7.86, 55.82) | 75.81 | 4.39(3.12) | 20.9(9.19) |
| 59 | gastrointestinal disorders | abnormal faeces | 3 | 5.34(1.72, 16.56) | 5.33(1.71, 16.61) | 10.56 | 2.41(1) | 5.33(2.07) |
| 60 | gastrointestinal disorders | infrequent bowel movements | 3 | 18.17(5.85, 56.43) | 18.16(5.83, 56.6) | 48.55 | 4.18(2.76) | 18.13(7.02) |
| 61 | vascular disorders | peripheral coldness | 87 | 108.73(87.81, 134.63) | 106.3(85.68, 131.88) | 8978.65 | 6.72(6.41) | 105.16(87.94) |
| 62 | vascular disorders | cyanosis | 24 | 30.66(20.51, 45.84) | 30.48(20.6, 45.11) | 682.3 | 4.93(4.36) | 30.39(21.71) |
| 63 | vascular disorders | pallor | 10 | 5.96(3.2, 11.09) | 5.95(3.18, 11.14) | 41.14 | 2.57(1.72) | 5.94(3.54) |
| 64 | vascular disorders | kawasaki's disease | 4 | 269.8(99.85, 729.02) | 269.52(99.19, 732.34) | 1041.18 | 8.03(6.75) | 262.26(114.16) |
| 65 | vascular disorders | vasoconstriction | 3 | 70.88(22.76, 220.77) | 70.82(22.72, 220.73) | 205.02 | 6.14(4.71) | 70.32(27.18) |
| 66 | investigations | blood pressure decreased | 15 | 3.76(2.27, 6.25) | 3.75(2.25, 6.24) | 30.29 | 1.91(1.2) | 3.75(2.45) |
| 67 | investigations | heart rate decreased | 12 | 4.99(2.83, 8.8) | 4.98(2.82, 8.79) | 38.18 | 2.32(1.53) | 4.98(3.1) |
| 68 | investigations | body temperature decreased | 4 | 5.7(2.14, 15.21) | 5.7(2.14, 15.19) | 15.49 | 2.51(1.24) | 5.7(2.51) |
| 69 | investigations | myocardial necrosis marker increased | 3 | 23.7(7.63, 73.63) | 23.68(7.6, 73.81) | 65.02 | 4.56(3.15) | 23.63(9.15) |
| 70 | investigations | respiratory rate decreased | 3 | 17.57(5.66, 54.56) | 17.56(5.63, 54.73) | 46.76 | 4.13(2.72) | 17.53(6.79) |
| 71 | cardiac disorders | bradycardia | 48 | 14.95(11.24, 19.88) | 14.78(11.23, 19.45) | 616.05 | 3.88(3.48) | 14.75(11.63) |
| 72 | cardiac disorders | atrioventricular block | 5 | 11.29(4.69, 27.14) | 11.27(4.67, 27.23) | 46.75 | 3.49(2.34) | 11.26(5.4) |
| 73 | cardiac disorders | cardiac dysfunction | 5 | 16.9(7.03, 40.66) | 16.88(6.99, 40.78) | 74.57 | 4.07(2.92) | 16.85(8.08) |
| 74 | skin and subcutaneous tissue disorders | skin discolouration | 13 | 4.28(2.48, 7.38) | 4.27(2.47, 7.39) | 32.56 | 2.09(1.34) | 4.27(2.71) |
| 75 | skin and subcutaneous tissue disorders | dermatitis diaper | 7 | 149.21(70.69, 314.99) | 148.95(70.73, 313.69) | 1013.13 | 7.2(6.19) | 146.71(78.52) |
